# Experimental validation of non-uniformity effect of the radial electric field on the edge transport barrier formation in JT-60U H-mode plasmas

**DOI:** 10.1038/srep30585

**Published:** 2016-08-02

**Authors:** K. Kamiya, K. Itoh, S.-I. Itoh

**Affiliations:** 1 National Institutes for Quantum and Radiological Science and Technology (QST), Naka, Ibaraki-ken 311-0193, Japan; 2 National Institute for Fusion Science (NIFS), Toki, Gifu 509-5292, Japan; 3 Research Institute for Applied Mechanics, Kyushu University, Kasuga, Kasuga koen 6-1, 816-8580, Japan; 4 Research Center for Plasma Turbulence, Kyushu University, Kasuga 816-8580, Japan

## Abstract

The turbulent structure formation, where strongly-inhomogeneous turbulence and global electromagnetic fields are self-organized, is a fundamental mechanism that governs the evolution of high-temperature plasmas in the universe and laboratory (e.g., the generation of edge transport barrier (ETB) of the H-mode in the toroidal plasmas). The roles of inhomogeneities of radial electric field (*E*_*r*_) are known inevitable. In this mechanism, whether the first derivative of *E*_*r*_ (shear) or the second derivative of *E*_*r*_ (curvature) works most is decisive in determining the class of nontrivial solutions (which describe the barrier structure). Here we report the experimental identification of the essential role of the *E*_*r*_-curvature on the ETB formation, for the first time, based on the high-spatiotemporal resolution spectroscopic measurement. We found the decisive importance of *E*_*r*_-curvature on ETB formation during ELM-free phase, but there is only a low correlation with the *E*_*r*_-shear value at the peak of normalized ion temperature gradient. Furthermore, in the ELMing phase, the effect of curvature is also quantified in terms of the relationship between pedestal width and thickness of the layer of inhomogeneous *E*_*r*_. This is the fundamental basis to understand the structure of transport barriers in fusion plasmas.

Since the discovery in ASDEX tokamak^1^, the high confinement mode (H-mode) was studied on many tokamaks, leading to the “baseline” operation scenario for ITER^2^. Theoretical work predicted that the edge radial electric field, *E*_*r*_, should play an important role in the mechanism of the L-H transition^3^. The key idea is the self-organized dynamics of strong radial electric field and suppression of transport^3^,^4^,^5^, as is summarized in^6^. According to theoretical predictions, extensive measurements for the ion density, temperature, and poloidal/toroidal flows at the plasma peripheral region were performed by means of spectroscopic method^7^,^8^, and experimental verifications on many devices were also performed^9^,^10^,^11^, exhibiting the localized *E*_*r*_ and significant reduction in a plasma turbulence level. It is conventionally believed that the shear of ***E×B*** drift velocity (*i.e.* first derivative of *E*_*r*_) is the main parameter that is responsible for suppression of turbulence^5^,^12^,^13^,^14^,^15^. In addition, the important role of the curvature of radial electric field (*i.e.* second derivative of *E*_*r*_) in suppressing the turbulence and turbulent transport has also been pointed out^16^,^17^,^18^,^19^,^20^. However, there has been no quantitative verification for both *E*_*r*_-shear and curvature effects on the ETB formation, simultaneously, due to lack of diagnostic that can withstand up to the second derivative estimation. Whether the first derivative or the second derivative works most in turbulence suppression is decisive in determining of class of nontrivial solutions (as has been theoretically pointed out^21^). The influence of the curvature of *E*_*r*_ has been more widely investigated when one considers the excitation of Zonal flows (ZFs) by the drift wave (DW) turbulence (see, a review^22^). The discrimination of roles of the shear and curvature of *E*_*r*_ in reducing turbulence is essential. This is because, these two represent the two fundamental processes in turbulent structure formation, i.e., (1) the enhanced dissipation of DW fluctuations and (2) the convergence of DW energy into coherent axial vector fields, respectively. It is also important for understanding the formation of transport barriers in toroidal confinement devices. For instance, the “curvature transition”, a kind of the internal transport barriers^23^, looks to appear in conjunction with the *E*_*r*_ curvature. The trapping of turbulence intensity in the trough of *E*_*r*_ 24 has also been observed^25^,^26^. Here we report the experimental identification of the essential role of inhomogeneities of *E*_*r*_ on the ETBs formation, for the first time, based on the high-spatiotemporal resolution spectroscopic measurement^27^,^28^,^29^, being to develop the quantitative examination of two independent (i.e. *E*_*r*_-shear and -curvature) effects on ETBs formation. It is shown that both the shear and curvature of *E*_*r*_ work, but the role of curvature is inevitable in the formation of transport barrier. This discovery has a fundamental impact on our understanding of structure formation in magnetized plasmas, in addition to a substantial impact on our predictability of performance of future burning plasma experiments, since the over-all performance of toroidal plasma fusion device critically depends on the property of the ETB. For instance, the uncertainty in the fusion power amplification factor of ITER was estimated as about factor 10, and the main origin for which is the variance in the prediction of the width of the ETB^30^.

## Results

### Theoretical model

The model of turbulence intensity, in the presence of inhomogeneous radial electric field, was assessed based on the model formula for the effect of electric field shear^5^,^12^,^13^ and that of electric field curvature in^31^,^32^, and is used in the experimental test here as;


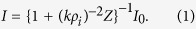


where the parameters are as follows:


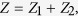



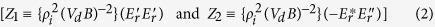


*I* is the turbulence intensity (mean square of fluctuation velocity normalized to the diamagnetic speed), *ρ*_*i*_ is the ion gyro-radius, *k* is a typical wavenumber for the plasma turbulence, *V*_*d*_ is the diamagnetic velocity (*V*_*d*_ ≡ T/*eaB*), *e*: elementary charge, *a*: characteristic mean-scalelength, *B*: total magnetic field), 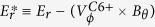 is the modified radial electric field subtracting the toroidal rotation component (estimated by the product of the toroidal rotation velocity for fully tripped carbon impurity ions, 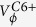, and poloidal magnetic field, *B*_*θ*_), and *I*_*0*_ is the mean intensity in the limit of *Z* = 0. The term *Z*_1_ and *Z*_2_ denote the effects of *E*_*r*_-shear (*i.e.* first derivative of *E*_*r*_ defined as 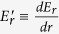)^5^,^12^,^13^ and *E*_*r*_-curvature (*i.e.* second derivative of *E*_*r*_ defined as 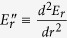)^31^, respectively. [Note that the correction for the effect of toroidal flow is necessary only for the *E*_*r*_ value in the *Z*_2_ term. This is because the force by turbulent Reynolds stress 
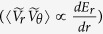
 is mainly in the poloidal direction, and its work is the product of force and poloidal velocity (not ***E*** × ***B*** velocity) as 
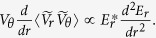
] The term *Z*_2_ has its sign dependence.

In association with the turbulence reduction by the inhomogeneous *E*_*r*_, the temperature gradient is enhanced. If one employs the gyroBohm dependence of local thermal diffusivity, which is proportional to the temperature gradient and turbulent intensity, the heat flux is proportional to the turbulence intensity multiplied by (*∇T*)^2^. For fixed heat flux, one can evaluate the enhancement degree of the ion temperature gradient in terms of Z value as;


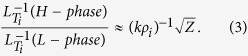


Here, the 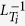 is the normalized ion temperature gradient 
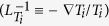
. The characteristic value of the wave number has been considered to be *k*ρ_i_ ~ 0.1 – 0.3 33. In the present examination, the wavelength of fluctuations is not measured, so that the proportionality between the ratio of gradient scale lengths and the parameters Z_1_, *Z*_*2*_, and *Z* are investigated. When the turbulent transport is reduced to the level of neoclassical transport, further reduction of turbulence level is less effective in increasing the ion temperature gradient.

### Experimental results (I): Temporal dynamics in ELM-free phase

**Figure 1:**
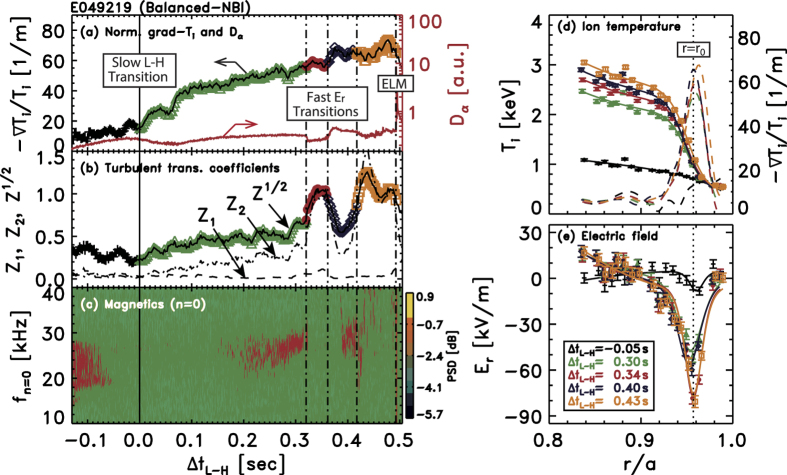
Temporal evolutions for (**a**) normalized ion temperature gradient 
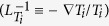
 and *D*_*α*_ emission, (**b**)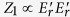, 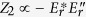 and square root of *Z*(≡*Z*_1_ + *Z*_2_) as defined in Eq. 2, and (**c**) Frequency and time resolved spectrogram for n = 0 component in the magnetic fluctuation detected by the sum of eight channel saddle loop arrays (toroidal). Radial profiles for (**d**) ion temperature and its inverse scale-length (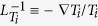), and (e) radial electric field are also shown. Vertical dotted line (*r* ≡ *r*_0_) for (**d**) and (**e**) corresponds to the location at which the 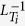 has a local peak value. Temporal evolutions for 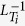, Z^1/2^, Z_1_, and Z_2_ are evaluated at r = r_0_.

In examining the effect of non-uniformity of *E*_*r*_ on the ETBs formation, Fig. 1 (discharge E049219 with balanced-NBI heating) exhibits the illuminating data that is suitable for model validation^27^. The high spatial resolution allows the observation of the shear and curvature of *E*_r_ in the transport barrier, so that their role on ETBs formation can be analyzed separately. This discharge shows a long period of secular increase in the edge ion temperature gradient after a “soft” L-H transition having a longer time-scale (a few hundred milliseconds) in comparison with a conventional “hard” one having shorter time-scale (*e.g.* a few milliseconds or less) seen in many tokamaks. This is followed by a violent phase, where the edge plasma bifurcates between a strong-*E*_*r*_ and a weak-*E*_*r*_ state (occurred at the normalized electric field condition of 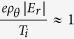
28, where the *ρ*_*θ*_ is the ion poloidal gyro-radius), until it finally settles to the conventional H-mode. The time period of 0 < Δt_L-H_ < 0.3 s in Fig. 1(a) is considered to be quasi-stationary for turbulence evolution and is suitable in evaluating the influence of inhomogeneous *E*_*r*_.

Looking at the slow L-H transition phase (0 < Δt_L-H_ < 0.3 s), we found that the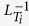 increases as the 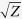 value becomes more positive, as shown in Fig. 1(a,b). The main contribution to the change of *Z* is from *Z*_2_, and *Z*_1_ remains small during the growth of the temperature gradient. An important caveat to this discussion is that there is only a low correlation between *Z*_*1*_ and 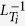 at which 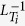 has a peak value in the pedestal (defined by *r* ≡ *r*_0_ as illustrated in Fig. 1d), while the 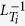 value at *r* = *r*_*0*_ becomes large as the *Z*_*2*_ value at *r* = *r*_*0*_ increases. This shows the relative importance of nonzero second derivative effect 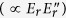 with its sign dependence for the turbulent suppression rather than that of the first derivative effect 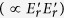, especially for the ETBs region around *r* = *r*_*0*_ (the effect of *E*_*r*_-shear/curvature on other remaining region in the pedestal, such as *r* ≤ *r*_*0*_, will be discussed later). It should be noted that the location at which the 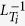 has a local peak value (i.e. *r* = *r*_*0*_) is closely related to that at which the *E*_*r*_ (and/or its curvature) has a local peak value as shown in Fig. 1(d,e), where the *E*_*r*_-shear has almost zero value. Indeed, this fact is confirmed by a quantitative comparison for various pedestal structures obtained in JT-60U H-mode plasmas with different angular momentum injections (*e.g.* co- and counter-NBI plus perpendicular-NBI heating)^29^.

It is interesting that a uniform toroidal MHD oscillation (i.e. *n* = 0) seems to be associated with the multi-stage *E*_*r*_-transitions during ELM-free H-phase as shown in Fig. 1(c). Since the frequency dependence of this MHD mode (*f*_*GAM*_ ≈ *C*_*s*_/2*πR*) is likely to that of the predicted frequency for Geodesic Acoustic Mode (GAM) known as a high-frequency blanch of Zonal flow (ZF), this observation can also support the hypothesis of ZF (and/or *E*_*r*_-curvature) suppression of turbulence even in the H-mode plasmas.

**Figure 2:**
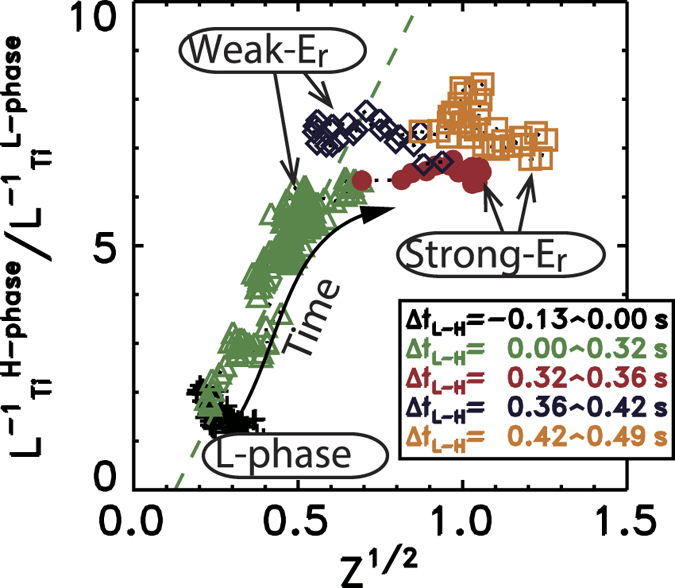
Relationship between the 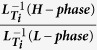 and 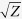 (
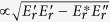
 as defined in Eq. 2) at which the 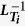 (and/or Er) has a local peak value for discharge E049219 (balanced-NBI). The line (dashed) Y = −1.7 + 13.4 X is drawn, where Y is 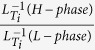 and X is 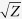, in which we use X and Y data for the slow L-H transition phase (*Δ*t_L-H_ = 0.0 – 0.32 sec).

The quantitative comparison between the temperature gradient and inhomogeneity effect of electric field is illustrated in Fig. 2. In the slow L-H transition phase (*Δt*_*L*-*H*_ = 0.0–0.32 *s*) there is a good correlation between the 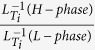 and 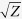 at *r* = *r*_*0*_. The enhancement of the gradient (by the factor of a few times) appears in the range of 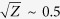. The observed proportionality between increased gradient and 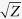 is to hold approximately 
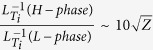
. It is in the range of expectation with the assumption of *k*ρ_i_ ~ 0.1–0.3.

The relationship between 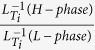 and the parameter *Z* shows a more complex (*i.e.* non-linear) behaviour after * Δt*_*L*-*H*_ = 0.32 *s*, where 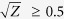. During the phase *Δt*_*L*-*H*_ = 0.32–0.36 *s*, a fast (within milliseconds) and strong drop in 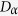 emission can be seen (Fig. 1a), while the increase in the 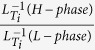 seems to be lower (up to about 10% or less). This suggests that there are additional mechanisms in the final stage of the H-mode transition in addition to the mechanism included in Eqs 1 and 2. At *Δt*_*L*-*H*_ = 0.36 *s*, the 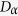 emission increases rapidly, and dramatic changes in the edge *E*_*r*_ structure from strong-*E*_*r*_ to weak-*E*_*r*_ are visible. These changes are similar to those at *Δt*_*L*-*H*_ = 0.42 *s*, but the changes are in opposite direction. However, this does not seem to be the same as the H-L backward transition commonly associated with the L-mode but, rather, this phase (*Δt*_*L*-*H*_ = 0.36–0.42 *s*) seems to be similar to the phase *Δt*_*L*-*H*_ = 0.0–0.32 *s*. After *Δt*_*L*-*H*_ = 0.42 *s*, the plasma changes again, with characteristics similar to the phase *Δt*_*L*-*H*_ = 0.32–0.36 *s*. This phase continues until the first ELM onset at *Δt*_*L*-*H*_ = 0.49 *s*.

The question why the dependence of 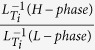 on 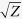 at *r* = *r*_*0*_ is likely to be non-linear, especially for the later ELM-free H-phase (during *Δt*_*L*-*H*_ = 0.32–0.36 *s* and *Δt*_*L*-*H*_ = 0.42–0.49 *s*), requires further studies: Let us explain a hypothesis to understand this stimulating observation. Before the jump of electric field occurs, the suppression of turbulence is strong enough so that the ion thermal transport is reduced to the level of the neoclassical transport. The abrupt bifurcations in the edge *E*_*r*_ to larger values (say, Z ~ 1), suppress the turbulence as was shown in^27^, so as to induce the jumps in 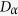 emission. However, the ion thermal transport is less sensitive, owing to the remaining neoclassical thermal transport for ions. Indeed, we confirm that the dependence of 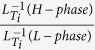 on 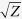 at *r* = *r*_*0*_ seems to be almost linear for the slow L-H transition phase having weak *E*_*r*_ (*Δt*_*L*-*H*_ = 0.0–0.32 *s*), including another phase having weak *E*_*r*_ (*Δt*_*L*-*H*_ = 0.36–0.42 *s*), as expected from Eq. 3.

### Experimental results (II): Spatial dynamics in ELMing phase

**Figure 3:**
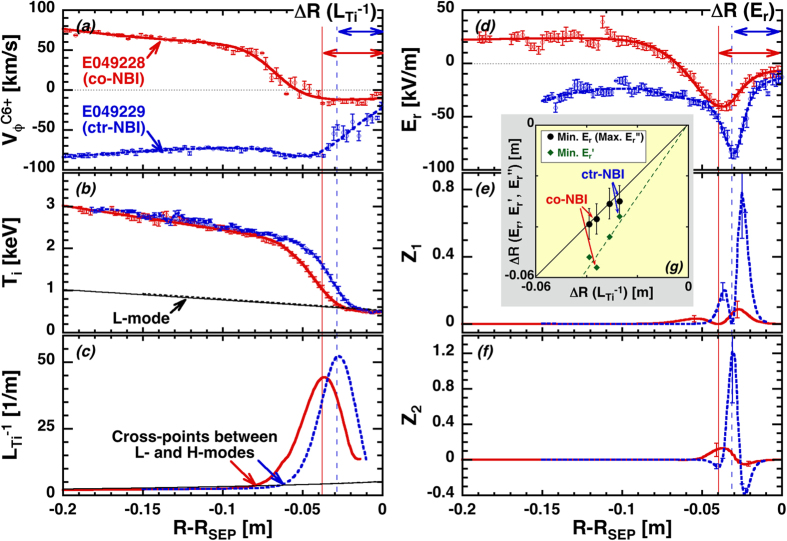
Radial profiles for (**a**) toroidal rotation for fully-stripped carbon impurity ions, (**b**) ion temperature, *T_i_*, (**c**) inverse ion temperature scale-length 
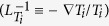
, (d) radial electric field, *E*_*r*_, (**e**) 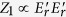 and (**f**) 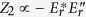 as defined in Eq. 2 in the discharges E049228 (co-NBI) and E049229 (ctr-NBI). Vertical lines (solid line: co-NBI, dashed line: ctr-NBI) are drawn for the locations at which the 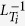 and *E*_*r*_ have a local peak values [defined as 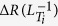 and 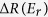, respectively], exhibiting a strong correlation between 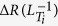 and 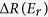 for various pedestal structures obtained in JT-60U H-mode plasmas with different angular momentum injections as summarized in (**g**).

Experimental data presented in Fig. 3 exhibit the highest quality data that is also suitable for model validation in a different perspective, especially for the relationship between pedestal width and thickness of the inhomogeneous edge *E*_*r*_ layer. These data are determined from multiple and reproducible Edge Localized Modes (ELMs) cycles for co- and counter-NBI discharges, mapping them onto a single-time basis, as defined by the time of the measurement relative to the ELMs for improved statistics to assess the temporal behavior (in particular inter-ELM phase) of the measurements^29^.

As shown in Fig. 3(c), the pedestal width (evaluated at the cross-point of the 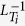 profiles between L- and H-mode phases) seems to be expanded up to *R-R*_*SEP*_ ≥ −0.08 m for co-NBI case, while it becomes narrower up to *R-R*_*SEP*_ ≥ −0.06 m for counter-NBI case. Looking at Fig. 3(e,f), we found that non-zero Z_1_ layer (i.e. impact of the 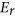-shear on turbulence suppression is effective) seems to be restricted in a range of *R-R*_*SEP*_ ≥ −0.07 m (co-NBI) and −0.05 m (counter-NBI), respectively, indicating about 1 cm narrower than that of the pedestal width. Furthermore, there is Z_2_ layer of the negative value (contributing turbulence enhancement) around *R*-*R*_*SEP*_ ≈ −0.04 m in the counter-NBI case.

It should be noted that we found a strong correlation between the location at which the 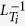 and *E*_*r*_ (and/or its curvature) had a local peak values for both co- and counter-NBI cases as shown in solid line on Fig. 3(g). On the other hand, the location at which the *E*_*r*_-shear had a local peak value shift inwards from the radius where the 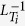 had a local peak value. The difference of the positions of the peak of 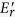 and 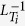 is confident, beyond the error bar, although we could see a linear correlation (i.e. direct proportion excepting its proportionality coefficient) between them. This conclusion holds for both co- and counter-NBI cases as shown in dotted line on Fig. 3(g). The most important point on this discussion is that we could confirm the conclusion in the ELM-free phase [i.e. the relative importance of nonzero second derivative effect 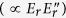 for the turbulent suppression rather than that of the first derivative effect 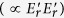], being described in Fig. 1d,e] even in the ELMing phase much more accurately.

**Figure 4:**
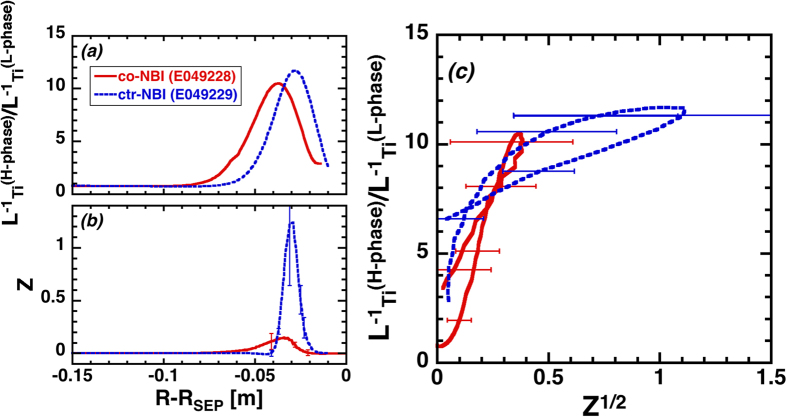
Radial profiles for (**a**) ratio of the inverse ion temperature scale-length 
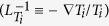
 in H- to L-phases, 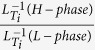, (**b**) effect of an inhomogeneous radial electric field on the turbulence suppression predicted by the *E*_*r*_-bifurcation model, 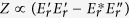 as defined in Eq. 2 in the discharges E049228 (co-NBI) and E049229 (counter-NBI). Relationship between 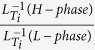 versus square root of Z is also shown in (**c**).

As a result, we found the Z-profile had almost non-zero value in the pedestal region (at least for its full width at half maximum of 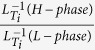 value) for both co- and counter-NBI cases as shown in Fig. 4(a,b). Furthermore, we confirmed that the dependence of the 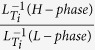 value on 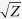 exhibited a similar trend for both co- and counter-NBI cases as illustrated in Fig. 4(c). It should be noted that the relationship between those factors obtained in the ELM-free phase at a fixed location (i.e. *r* = *r*_*0*_ defined for discharge E049219) shows a similar nature to that obtained even in the ELMing phase (at the time of ELM-onset) for various locations (discharges E049228 and E049229).

This result is not self-evident, since the *Z* value in the latter case (i.e. spatial structure in the ELMing phase) contains both *E*_*r*_-shear and *E*_*r*_-curvature effects, while the *E*_*r*_-curvature effect is dominant contributor for *Z* value in the former case (i.e. temporal trajectory in the ELM-free phase).

It is also noted that the improved energy confinement region (*e.g.* 
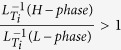
) is observed even in the region near the pedestal top, where Z value is small. The smooth connection phenomena between L-mode region (*e.g.* around the pedestal-top) and H-mode pedestal (*e.g.* well inside the pedestal-top) results in an up-shift of the curve in Fig. 4(c), in comparison with that seen in Fig. 2.

### Discussion and Summary

In summary, we revisited the studies of paradigm of shear suppression of turbulence as the mechanism for the ETBs formation with an improved diagnostic for the edge radial electric field from the high resolution measurement on JT-60U tokamak, examining the effects of both shear and curvature, comparing with *E*_*r*_-bifurcation model. Focusing on the relationship between the normalized ion temperature gradient and non-uniform radial electric field structures, we found that *E*_*r*_-curvature effects are essential, (not only the *E*_*r*_-shear) in forming the ETBs. Recalling that the non-uniform *E*_*r*_ effect presented in this letter should have 2^nd^ order effect of ***O***(*E*_*r*_^*2*^), the Z profile is localized strongly around the location, at which the 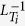 has a local peak value in the pedestal, and hence inhomogeneous electric field seen in the H-mode pedestal makes it possible to have an impact on the turbulence suppression even at the lower edge of the pedestal structure.

Indeed, this effect may be able to exude toward the pedestal top (and/or bottom) due to its 2^nd^ order effect, leading a new prediction of the pedestal width scaling (and/or providing an possible physics understanding for its scaling using non-dimensional parameters in many tokamas). It should be noted that the thickness in the ***E*** × ***B***shear layer was roughly scaled by a few times of the 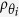 in the previous publication based on a poor spatial resolution^34^. It is also suggested that there is additional mechanism to regulate the temperature gradient in the fully developed H-mode plasmas^35^.

## Methods

### JT-60U

The JT-60U tokamak is a single null divertor tokamak device having the plasma major radius, R_P_ = 3–3.5 m, the plasma minor radius, a_P_ = 0.6–1.1 m and the maximum toroidal magnetic field, B_T_ ≤ 4 T at R = 3.32 m. We performed the NBI heating experiments by comparing the external momentum input directions between co-, balanced- and counter-NBI cases under a matched plasma shape condition. The plasma current, I_P_, was 1.6 MA, and the toroidal magnetic field, B_T_, was 3.9 T. The corresponding safety factor at the 95% flux surfaces, q_95_, was thus 4.2. The elongation, κ, and triangularity, δ, were 1.47 and 0.36, respectively, and the total plasma volume was 57 m^3^.

### Charge eXchange Recombination Spectroscopy (CXRS)

In the study of the JT-60U tokamak for the fiscal year (FY) 2007–2008 experimental campaign, we measured the radial profiles for the density, temperature, and poloidal/toroidal plasma flows of fully stripped carbon impurity ions by means of the Charge eXchange Recombination Spectroscopy (CXRS) diagnostic method with fast time resolution (up to 400 Hz) at 59 spatial points (23 toroidal and 36 poloidal viewing chords). With regard for determining the E_r_ structure at the pedestal region, we measured the pressure gradient, and plasma velocity perpendicular to the magnetic field, and the E_r_ was evaluated by the radial force balance equation.

## Additional Information

**How to cite this article**: Kamiya, K. *et al.* Experimental validation of non-uniformity effect of the radial electric field on the edge transport barrier formation in JT-60U H-mode plasmas. *Sci. Rep.* **6**, 30585; doi: 10.1038/srep30585 (2016).
